# MPL mutations are over-represented in patients with dual driver-mutated myeloproliferative neoplasms: an Irish cohort study

**DOI:** 10.1016/j.htct.2026.106495

**Published:** 2026-07-09

**Authors:** Laura Kearney, Claire Andrews, Rachel Brodie, Ezzat Elhassadi, Helen Enright, Kamal Fadalla, Michael Fay, Catherine Flynn, Anne Fortune, Andrew Hodgson, Senthil Kumar, Kanthi Perera, Jeremy Sargent, Liam Smyth, Stephen E. Langabeer

**Affiliations:** a Cancer Molecular Diagnostics, St. James’s Hospital, Dublin, Ireland; b Department of Haematology, St. Vincent’s University Hospital, Dublin, Ireland; c Department of Haematology, Cork University Hospital, Cork, Ireland; d Department of Haematology, University Hospital Waterford, Waterford, Ireland; e Department of Haematology, Tallaght University Hospital, Dublin, Ireland; f Department of Haematology, Mater Misericordiae University Hospital, Dublin, Ireland; g Department of Haematology, St. James’s Hospital, Dublin, Ireland; h Department of Haematology, Sligo University Hospital, Sligo, Ireland; i Department of Haematology, Midlands Regional Hospital, Tullamore, Ireland; j Department of Haematology, Beaumont Hospital, Dublin, Ireland

Dear Editor

Singular mutations in either *JAK2, CALR* or *MPL* genes are the most frequent drivers of the myeloproliferative neoplasms (MPN) with variants in other myeloid-associated genes contributing to disease phenotype and clinical course. MPN molecular diagnostic algorithms can be either sequential or simultaneous with each technology employed possessing its own sensitivities, specificities and clinical applicability [1]. An increasingly recognised, minor subgroup of MPN patients, particularly those with essential thrombocythemia (ET) and primary myelofibrosis (PMF), harbor two driver mutations [2]. Prior evidence suggests a second driver mutation occurs more commonly in low variant allele frequency (VAF) *JAK2* V617F-mutated MPN with the presence of two mutations affecting the phenotype such as a higher platelet count [3,4].

In order to evaluate the incidence and molecular diversity of the dual driver phenomenon in the Irish MPN population, an audit was performed on all requests received at a center for molecular diagnosis of MPN. Mutations in *JAK2* (exons 12 and 14), *CALR* (exon 9), and *MPL* (exon 10) were identified using a previously described single-molecule, molecular inversion probe-based next-generation sequencing (NGS) methodology [5,6]. From March 2023 to November 2025, 1275 consecutive, new patients were identified with either *JAK2* V617F (n = 1011; 79.3%), *JAK2* exon 12 (n = 12; 0.9%), *CALR* exon 9 (n = 168; 13.2%) or *MPL* exon 10 (n = 84; 6.6%) mutations. This cohort consisted of 665 females and 610 males with a median age of 68 years (range: 21–97 years). Of these 1275 patients, thirteen (1.0%) were identified with ET (n = 12) or PMF (n = 1) with two driver mutations. This group comprised eight females and five males with a median age at presentation of 71 years (range: 61–89 years) and a median presenting platelet count of 853 × 10^9^/L (range: 562–1244 × 10^9^/L) (Table 1). Of the 26 mutations in 13 patients, eleven (42.3%) were *JAK2* V617F, eleven (42.3%) were in *MPL* exon 10 and four (15.4%) were in *CALR* exon 9. Of the eleven patients with a *JAK2* V617F, seven (63.6%) had a VAF ≤5% (Table 1).

**Table 1:** Myeloproliferative neoplasm patients with dual driver mutations. Largest variant allele frequency (VAF) mutation first.

Case	Sex	Age (years)	MPN	Platelets (x 10^9^/L)	Mutation #1	VAF (%)	Mutation #2	VAF (%)
#1	F	64	ET	615	*MPL* p.(Trp515Leu)	27.0	*JAK2* p.(Val617Phe)	2.0
#2	F	84	ET	736	*JAK2* p.(Val617Phe)	47.0	*CALR* p.(Leu367Thrfs*46)	3.9
#3	F	71	ET	1147	*CALR* p.(Lys385Asnfs*47)	3.5	*JAK2* p.(Val617Phe)	1.1
#4	F	70	ET	958	*JAK2* p.(Val617Phe)	6.1	*MPL* p.(Trp515Leu)	2.7
#5	M	67	ET	787	*CALR* p.(Leu367Thrfs*46)	30.0	*JAK2* p.(Val617Phe)	3.2
#6	F	61	ET	1113	*MPL* p.(Trp515Leu)	31.0	*JAK2* p.(Val617Phe)	1.5
#7	F	89	ET	1244	*MPL* p.(Trp515Arg)	4.4	*JAK2* p.(Val617Phe)	1.1
#8	F	85	ET	853	*MPL* p.(Trp515Arg)	9.6	*MPL* p.(Ser505Asn)	9.3
#9	F	86	ET	1100	*JAK2* p.(Val617Phe)	7.2	*MPL* p.(Ser505Asn)	5.4
#10	M	70	PMF	857	*JAK2* p.(Val617Phe)	17.0	*MPL* p.(Trp515Leu)	2.8
#11	M	69	ET	532	*MPL* p.(Trp515Leu)	16.0	*JAK2* p.(Val617Phe)	2.3
#12	M	72	ET	624	*MPL* p.(Trp515Leu)	2.5	*CALR* p.(Glu371Metfs*42)	2.3
#13	M	81	ET	795	*MPL* p.(Trp515Ala)	11.0	*JAK2* p.(Val617Phe)	3.4

In this European cohort, 1% of MPN patients presented with dual driver mutations, similar to the recently described rate of 1.5% [7]. The association of dual driver mutations in patients with a low *JAK2* V617F VAF is also confirmed [8,9]. In agreement with a proposal that a platelet count higher than 700 × 10^9^/L acts as a general discriminator for identifying dual driver mutations [4], only three of thirteen patients presented with a lower platelet count. The relatively small size and short follow-up of this cohort preclude any clinical correlation. In MPN patients with a single driver mutation, *MPL* exon 10 mutations are present in approximately 5% and 9% of patients with ET and PMF respectively with the W515L allele being most prevalent. In contrast, *MPL* exon 10 mutations account for >40% of variants in this dual-driver population; these include the less common *MPL* S505N, W515R, and W515A mutations. This study provides confirmation of the prevalence of this molecular subtype in a national MPN cohort and demonstrates a distinct pattern of *MPL* mutations in dual driver MPN. This brief audit further underscores the molecular and clonal complexity of this subset of MPN patients.

## Funding

This research received no specific grant from any funding agency in the public, commercial, or not-for-profit sectors.

## Ethical approval

This non-interventional, observational study was performed under routine standard of care procedures in accordance with the 1964 Helsinki declaration and its later amendments. Informed consent was obtained from participants at the referring centers.

## Data availability

The data that support the findings of this study are available from the corresponding author upon reasonable request.

## Conflicts of interest

All authors declare no potential conflicts of interest with respect to the research, authorship, and/or publication of this article.
